# The Programming of Antioxidant Capacity, Immunity, and Lipid Metabolism in Dojo Loach (*Misgurnus anguillicaudatus*) Larvae Linked to Sodium Chloride and Hydrogen Peroxide Pre-treatment During Egg Hatching

**DOI:** 10.3389/fphys.2021.768907

**Published:** 2021-10-28

**Authors:** Mengya Wang, Wenyu Xu, Jiahong Zou, Shuaitong Li, Zixi Song, Feifei Zheng, Wei Ji, Zhen Xu, Qingchao Wang

**Affiliations:** ^1^Department of Aquatic Animal Medicine, College of Fisheries, Huazhong Agricultural University, Wuhan, China; ^2^Ocean University of China, Qingdao, China

**Keywords:** fish egg hatching, hydrogen peroxide, sodium chloride, programming, antioxidant capacity

## Abstract

Non-nutritional stress during early life period has been reported to promote the metabolic programming in fish induced by nutritional stimulus. Sodium chloride (NaCl) and hydrogen peroxide (H_2_O_2_) have been widely applied during fish egg hatching, but the influences on health and metabolism of fish in their later life remain unknown. In the present study, H_2_O_2_ treatment at 400mg/L but not 200mg/L significantly increased the loach hatchability and decreased the egg mortality, while NaCl treatment at 1,000 and 3,000mg/L showed no significant influences on the loach hatchability nor egg mortality. Further studies indicated that 400mg/L H_2_O_2_ pre-treatment significantly enhanced the antioxidant capacity and the mRNA expression of genes involved in immune response of loach larvae, accompanied by the increased expression of genes involved in fish early development. However, the expression of most genes involved in lipid metabolism, including catabolism and anabolism of loach larvae, was significantly upregulated after 200mg/L H_2_O_2_ pre-treatment. NaCl pre-treatment also increased the expression of antioxidant enzymes; however, only the expression of C1q within the detected immune-related genes was upregulated in loach larvae. One thousand milligram per liter NaCl pre-treatment significantly increased the expression of LPL and genes involved in fish early development. Thus, our results suggested the programming roles of 400mg/L H_2_O_2_ pre-treatment during egg hatching in enhancing antioxidant capacity and immune response of fish larvae *via* promoting fish early development.

## Introduction

The environmental and trophic conditions encountered at the early developmental period of animals have been confirmed to perform profound effects on the metabolism and physiology of individuals later in life, which is termed metabolic programming (when modifying metabolism; Lucas, 1998). Long-lasting modification in gene expression patterns is one of the most important biological mechanisms described in such case of adaptions, and it may persist later in life in the absence of the environmental stimulus that initiated them (George et al., 2012; Kongsted et al., 2014). In aquatic animals, including fish and shrimp, the concept of metabolic programming has been tested as well. Preliminary study in rainbow trout (*Oncorhynchus mykiss*) showed that only a strict nutritional stimulus had a minor programming effect on hepatic glucose metabolism (Geurden et al., 2007, 2014). Later studies indicated that an acute exposure to hypoxia alone (Liu et al., 2017a) or combined with an early nutritional stimulus, such as high-carbohydrate diet (Liu et al., 2017b), high dietary carbohydrate:protein ratios (Hu et al., 2018) induced obvious programming in the liver of juvenile rainbow trout. The hypoxic conditions resulted in the higher expression of *HIF-1α* which has been reported to modulate the nutrient metabolism (Menendez-Montes et al., 2021), antioxidant capacity (Lacher et al., 2018), and immune responses (Ni et al., 2020). However, the hypoxia may easily result in high mortality, and it is important to explore other non-nutritional stress. Due to the safety and friendly to human health and environment ecology, sodium chloride (NaCl) and hydrogen peroxide (H_2_O_2_) have been tested in the fry hatch of many fish species (Magondu et al., 2011). NaCl has been used effectively in aquaculture as antiparasitic agent (Schelkle et al., 2011; Dewi et al., 2018), growth-promoting agent in *Carassius auratus* (Imanpoor et al., 2012) and *Mugil liza* (Lisboa et al., 2015), and survival enhancing agent in *Pelecus cultratus* larvae (Kujawa et al., 2017), *Ictalurus punctatus*, *C. auratus*, *Morone saxatilis*, and *Acipenser oxyrinchus* (Altinok and Grizzle, 2001). Moreover, NaCl affects the embryonic development and larval vigor of *Epinephelus akaara* (Wang et al., 2002) and *Rhombosolea tapirina* (Hart and Purser, 1995). In one plateau species of loach, *Triplophysa (Hedinichthys) yarkandensis*, NaCl application with salinity at 4% resulted in the lowest deformity rate (Chen et al., 2016). H_2_O_2_ has received attention for its control of several fish pathogens and is recommended as a general disinfectant in aquaculture for treating aquaculture water and surface of tanks before introduction of fish (Avendaño-Herrera et al., 2006). H_2_O_2_ has been shown to promote the egg hatching rate of rainbow trout (Schreier et al., 1996; Barnes et al., 1998), channel catfish (*I. punctatus*; Small and Wolters, 2003), and *C. gariepinus* (Rasowo et al., 2007).

During multiple environmental challenges, free radical would be released, but the over-production of O_2_^−^ would cause oxidative damage to proteins, nucleic acids, and lipids (Kurien and Scofield, 2003). Thus, cellular antioxidant defenses system in fish and other animals are developed to scavenge the excessive reactive oxygen species (*ROS*; Pisoschi and Pop, 2015; Klein et al., 2017). Like hypoxia, H_2_O_2_ and NaCl treatment have also been proved to affect antioxidant capacity. Salinity or NaCl treatment significantly affected the mRNA expression and activity of antioxidant enzymes, including superoxide dismutase (*SOD*), glutathione S-transferase (*GST*), and glutathione (*GSH*) in multiple tissues of olive flounder (*Paralichthys olivaceus*; Kim et al., 2021), European seabass (*Dicentrarchus labrax*; Islam et al., 2020), *D. labrax,* and *Chanos Chanos* (Chang et al., 2021). Similarly, H_2_O_2_ exposure has also been reported to affect antioxidant capacity in common carp (*Cyprinus carpio*; Jia et al., 2020) and largemouth bass (*Micropterus salmoides*; Sinha et al., 2020). Besides antioxidant system, fish remains the first bony vertebrate to develop both innate and adaptive immunity which help themselves to defend against infected pathogens or other environmental challenges (Wang et al., 2019). The immune responses of European seabass and common carp (*C. carpio*) were significantly affected by different salinities (Islam et al., 2020) and H_2_O_2_ exposure (Jia et al., 2021), respectively. The programming effects on individuals of later life by environmental treatment or nutritional stimulus at early life stage mainly result from an alteration of the functional development of crucial organs (Pittman et al., 2013). It is well known that fish larvae along with the fertilized eggs grow very fast and experience significant changes in physiology; thus, they are very fragile and most susceptible to environmental stressors during fish ontogeny (Fuiman, 1983; Alvarez et al., 2021). The organs in the newly hatched fish larva are not well developed, and thus, it is not easy to do histological evaluation in fish larvae (Fuiman et al., 1999). The molecular methods *via* evaluating the relative mRNA expression levels of early development-related genes are useful and effective to systematically evaluate the influences of pre-treatment on the fry (Hu et al., 2018).

Dojo loach *Misgurnus anguillicaudatus* (Cantor 1842) is one of the important freshwater aquaculture species in China whose production has reached 367,428 tons by 2020 (Ministry of Agriculture and Rural Affairs of the People’s Republic of China, 2021) and can be used as a Chinese medicine for the treatment of hepatitis, carbuncles, inflammations, and cancers (Qin et al., 2002). The sustainable development of loach aquaculture industry relies on the stable loach fry supply, whose artificial breeding has been successfully overcome in recent years (Gao et al., 2014; Huang et al., 2015). However, the diseases resulting from microorganism infection or other environmental factors during fish hatchery have threatened the production of larval loach (Shamsi et al., 2021). The applications of antibiotics and insecticides have been seriously restricted in many countries (Holmström et al., 2003; Cabello, 2006; Shao et al., 2021), while no specific fish vaccine nor mature vaccination route is available for fish fry (Rojo-Cebreros et al., 2018; Wang et al., 2020), which seriously restricts the stable fish fry stocks. In the present study, NaCl and H_2_O_2_ were applied during loach egg hatching and the effects on the antioxidant capacity, immunity and lipid metabolism of fish larvae were evaluated as well as monitoring the early development-related genes.

## Materials and Methods

### Fish Stock and Egg Fertilization

Mature broodstock fish (average weight 18±2.1g), obtained from broodstock ponds, were selected and transferred to the hatchery. All fish were then acclimated in hatching tanks for 1day without feeding. To induce spawning, the selected female fish were injected with DOM (4mg/kg fish) and LRH-A2 (35μg/kg fish), and the male fish were injected with same reagents but half dosage. After 12h, the eggs were stripped into a dry bowl and fertilized with milt from a ripe male. After fertilization, the fertilized eggs were randomly counted into bottles with 100 eggs each. The individual hatching bottles were randomly assigned in triplicate to static bath treatments of given concentrations of either NaCl (1,000 and 3,000mg/L), H_2_O_2_ (200 and 400mg/L), and a control (nothing added) for 60-min exposure before being transferred to randomized compartments of the incubation tank for further incubation. The water temperature was controlled at 24–26°C and dissolved oxygen (DO) controlled at 7.5–7.8mg/L, which were monitored using an oxygen-temperature meter (model 55, YSI, Yellow Springs Ohio, United States).

### Egg Hatching and Hatchability Calculation

Loach larvae came out of the membrane after 24-h fertilization. Then, the hatching bottles were removed from the incubation tank. The numbers of live hatched larvae, dead hatched larvae, total dead eggs, and fungi-infected dead eggs were counted for the calculation of following parameters and then sent back to the incubation tank.


Hatchability (%) = The number of live hatched larvae/the total number of eggs * 100.

Fry mortality (%) = The number of dead hatched larvae/the total number of eggs * 100.

Egg mortality (%) = The number of total dead egg/the total number of eggs * 100.

Fungi-induced egg mortality (%) = The number of fungi-infected dead egg/the total number of eggs * 100.

Other factor-induced egg mortality (%) = The number of (total dead eggs excluding fungi-infected ones)/the total number of eggs * 100.

### Larviculture, RNA Extraction, and cDNA Synthesis

Loach larvae showed feed-hunting behavior at 4days after rupture, and then, larvae in all groups were fed with artemia for another 7days. At the end of feeding, all the loach larvae were collected and immediately frozen in liquid nitrogen and stored at −80°C before analysis.

The whole loach larvae were homogenized in TRIzol reagent (Invitrogen, Carlsbad, CA, United States) for RNA extraction according to the manufacturer’s recommendations. After RNA extraction procedures, the purity and concentration of RNA were monitored by NanoDrop 2000 spectrophotometer (Thermo scientific, United States), with their 260:280 ratios between 1.8 and 2.0. Additionally, 1.0% agarose gel electrophoresis was adopted to determine the integrity of RNA. The quantified RNA samples were then used for cDNA synthesis (Invitrogen, Carlsbad, CA, United States). Briefly, the potential existing genomic DNA was removed from the RNA samples with same amount using DNase. Then, 1μg of treated RNA was used for the synthesis of cDNA using the reverse transcriptase kit with oligo dT primers following manufacturer’s instructions.

### Quantitative RT-PCR

The synthesized cDNA was used for the quantitative real-time PCR (qPCR) analysis using the Eva Green 2×qPCR Master mix (ABM, Canada). qPCR was conducted on 7500 Real-time PCR system (Applied Biosystems, United States), with each PCR performed with triplicate samples and the cycling conditions set with 30s at 95°C, 1s at 95°C, and 10s at 58°C for 40cycles. In addition, a melt curve analysis was performed after amplification to verify the accuracy of each amplicon.

The relative quantification of the target genes involved in the antioxidant system [*SOD*, catalase (*CAT*), glutathione peroxidase (*GPx*), and metallothionein (*Mt*)], genes related to immune responses [*C1q*, *C3-1*, *C8b*, mannose-binding lectin-associated serine protease-1 (*MASP-1*), interleukin 15 receptor subunit alpha (*IL15Rα*), and heat shock protein 70 (*Hsp70*)], genes involved in lipid metabolism [carnitine palmitoyltransferase 1alpha (*Cpt1α*), lipoprotein lipase (*LPL*), fatty acid desaturase 2 (*Fads2*), and proliferator-activated receptor gamma (*PPARγ*)] and early development-related genes [spondin 1b (*spon1b*), intraflagellar transport protein 22 (*IFT22*), vascular endothelial growth factor Aa (*VEGFAa*), glutamate dehydrogenase (*gdh*), annexin A1a (*anxa1a*), vasoactive intestinal peptide (*VIP*), protein phosphatase 1 (*PP1*), and protein phosphatase 2A catalytic subunit beta isoform (*PP2AB*)] were determined *via* normalized against elongation factor 1-alpha (*EF1α*). Then, relative abundance of target genes was calculated by using the 2^−ΔΔ*C*t^ method. All primers used in the present study are shown in Table 1.

**Table 1:** Primers used in the present study.

*Gene*	Forward sequence	Reverse sequence
*Spon1b*	GTCGGACGGTTTCTGTAGGA	GAGGGTAAATCCACGAAAGTAAG
*IFT22*	TGGGATTGTGGAGGAGATTTC	AGTTTGCTCAGTTTTGGGGC
*VEGFAa*	TCTGCTCTATAACCCTCACCGC	GTCATTTTTGCTCTTCCCTCCT
*gdh*	TGCCTGTGTGACTGGTAAGCC	CCATAACGGTGAAGATAACGCA
*anxa1a*	TGCTGTGGTGAAATGTGCTG	AGTCTCCTTTGGTGTCGTCCT
*VIP*	GTCTCTTCACAAGCGGATACAG	TGGTCCTCCATCAAATCATCAC
*PP1*	GAGGACGGTTATGAGTTTTTTGC	GCTTTCTTCTCTGACGGCTTG
*PP2AB*	ACAGTCACACTTCTTGTTGCCCT	ATTTCCTCAAGCACTCGTCGTA
*SOD*	GACCATGCTGTGCAGAGTCGGATA	GGGCTGAAGGGACACTTGGGTAATA
*CAT*	GTGCTAAACCGAAACCCTGT	GCTGTTGGGGTAGTAGTTAGGAG
*GPx*	TCTAAATGAGGCAAGACCCCAGTA	CTCCCTTTAGGCTGTTCCTTCATC
*Mt*	GAAACGATACAGCAAAGGAACC	CTTACAAACGCATCCAGAGGC
*C1q*	TGCGTATGGTTGGCTTGTGGG	GAATAGGCGGTGAAGGAGAAAGAGTAGA
*C3-1*	TTTTCTATGATGCTGGTCTGATGTTTG	CGATGTACGTGGCTCGTCGTT
*C8b*	CCATGCCAGGGTTTCCGTTGT	CACCAGCATAGTAGCGGTTATCAAGC
*MASP-1*	ATAACTACATAGGTGGCTTCTACTGT	CCTCCTCTTGCTCAATGCGATACA
*IL15Rα*	GGAGCACAAGCAGACAAAAT	CTATGATTGATGTACTAGCTGGTTT
*Hsp70*	GGTCCTTCCAAGTCATCAG	GCAATCTCCTTCATCTTCAC
*Cpt1α*	CCATCTCTTCTGCCTCTAC	GCCACACCATAACCATCA
*LPL*	ACCTGGCTGTAACCTTCA	AACGGCATCATATCTCTGG
*Fads2*	CACAGGTTCGGCACTTACAC	TCGCATCTTCTCCAGCATAATG
*PPARγ*	TGGCTTTCACTATGGCGTTCA	GCATTTGTTGCGACTCTTCTTG
*EF1α*	TCAGCGCCTACATCAAGAAG	TTACGCTCAACCTTCCATCC

Spon1b, spondin 1b; IFT22, intraflagellar transport protein 22 homolog; VEGFAa, vascular endothelial growth factor Aa; gdh, glutamate dehydrogenase; anxa1a, annexin A1a; VIP, vasoactive intestinal peptide; PP1, protein phosphatase 1; PP2AB, protein phosphatase 2A catalytic subunit beta isoform; SOD, superoxide dismutase; CAT, catalase; GPx, glutathione peroxidase; Mt, metallothionein; MASP-1, mannose-binding lectin-associated serine protease-1; IL15Rα, interleukin 15 receptor subunit alpha; Hsp70, heat shock protein 70; Cpt1α, carnitine palmitoyltransferase 1alpha; LPL, lipoprotein lipase; Fads2, fatty acid desaturase 2; PPARγ, proliferator-activated receptor gamma; and EF1α, elongation factor 1-alpha.

### Statistical Analysis

All statistical analyses were performed using SPSS 17.0. Data were analyzed by one-way analysis of variance (ANOVA) followed by Tukey’s multiple range tests to determine the effects of NaCl and H_2_O_2_ on egg hatching and gene expression. Differences were considered significant when *p*<0.05. All data were expressed as mean±standard deviation of the mean (SD), except the specific statement.

## Results

### Effects of H_2_O_2_ and NaCl Treatment on Hatching Performances of Loach Larvae

**Figure 1:**
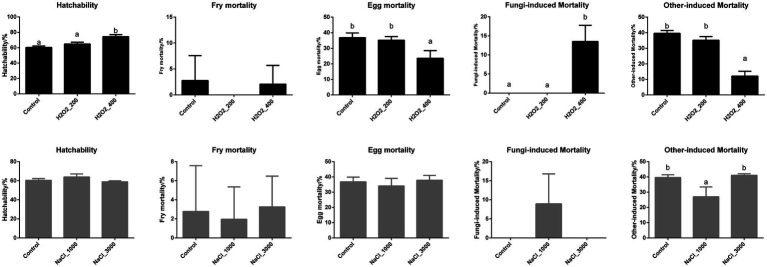
Effects of H_2_O_2_ (200, 400mg/ml) and NaCl (1,000, 3,000mg/ml) pre-treatment during egg hatching on the hatchability, fry mortality, egg mortality including fungi-induced mortality and other factor-induced mortality of loach.

The hatching performances including hatchability, larval mortality, egg mortality including fungi-induced mortality and other-induced mortality of loach after H_2_O_2_ and NaCl treatment are shown in Figure 1. Four hundred milligram per liter H_2_O_2_ treatment significantly increased larvae hatchability, while the larvae mortality showed no significant differences after H_2_O_2_ treatment. Additionally, the egg mortality was also significantly decreased after 400mg/L H_2_O_2_ treatment. However, the decreased egg mortality after 400mg/L H_2_O_2_ treatment was not due to fungi, but by other factors, as the fungi-induced egg mortality was even higher in 400mg/L H_2_O_2_ treatment group.

NaCl treatment showed no significant effects on larval hatchability nor fry mortality. Similarly, the total egg mortality along with the fungi-induced egg mortality was not affected by NaCl treatment. However, the other factor-induced egg mortality was decreased during 1,000mg/L NaCl treatment.

### Effects of H_2_O_2_ and NaCl Pre-treatment During Egg Hatching on the Expression of Genes Involved in Development of Loach Larvae

**Figure 2:**
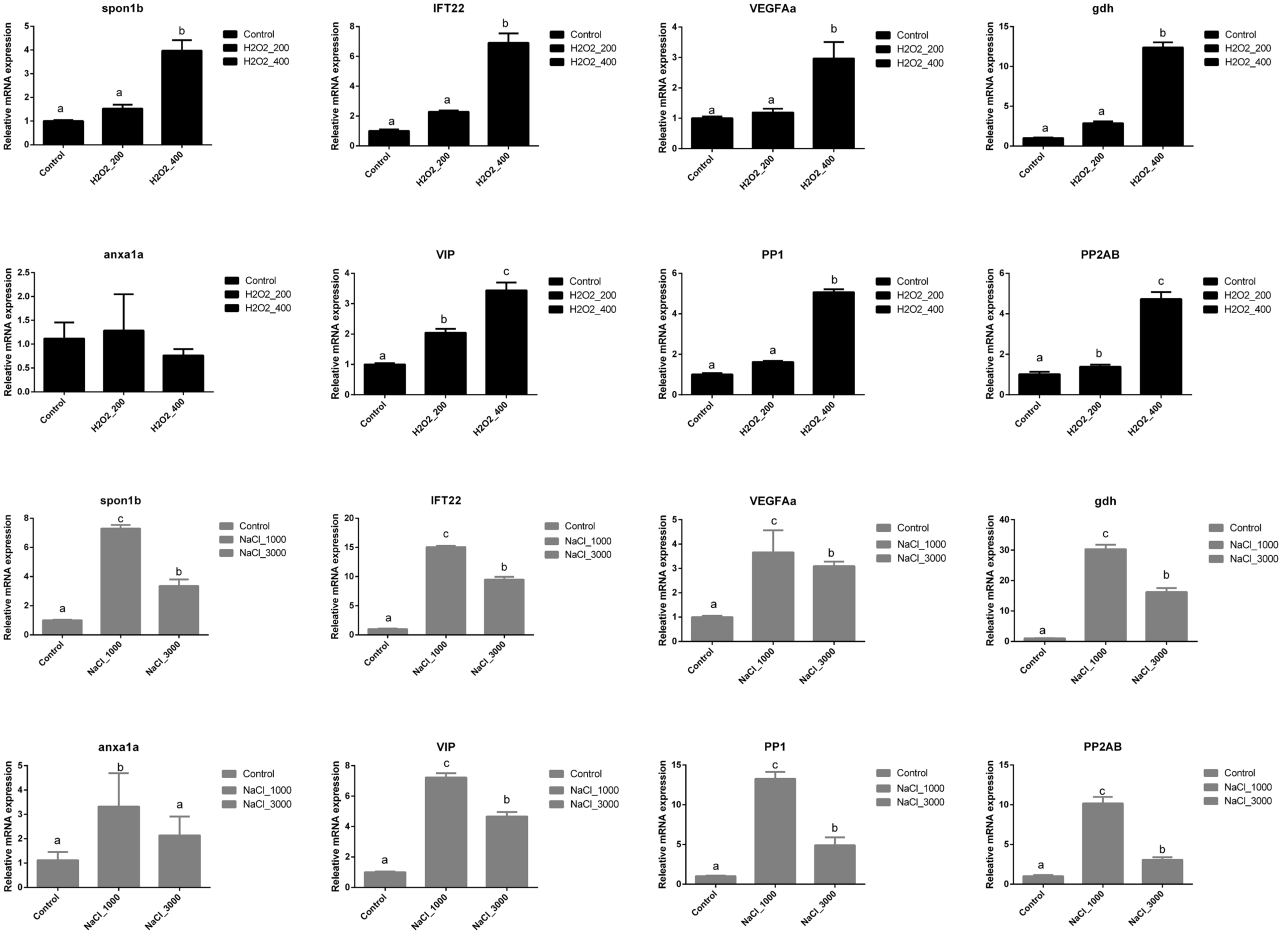
Effects of H_2_O_2_ (200, 400mg/ml) and NaCl (1,000, 3,000mg/ml) pre-treatment during egg hatching on the relative expression levels of genes involving early development including *spon1b, IFT22, VEGFAa, gdh, anxa1a, VIP, PP1,* and *PP2AB* in loach larvae.

Figure 2 indicated the influences of H_2_O_2_ and NaCl pre-treatment during egg hatching on the expression of early development-related genes of loach larvae. The expression of *spon1b*, *IFT22*, *VEGFAa*, and *PP2AB* was significantly upregulated after 400mg/L H_2_O_2_ pre-treatment. The expression of *gdh*, *VIP*, and *PP1* was significantly upregulated with the increased dosage of H_2_O_2_, and highest expression level was detected at 400mg/L H_2_O_2_ pre-treatment. No significant effects of H_2_O_2_ pre-treatment were detected on the expression of *anxa1a*.

The expression of *spon1b*, *IFT22*, *gdh*, *VIP*, *VEGFAa*, *PP1*, and *PP2AB* in loach larvae was significantly upregulated after NaCl pre-treatment; however, their expression levels were significantly higher at 1,000mg/L NaCl pre-treatment than those at 3,000mg/L NaCl pre-treatment. The expression of *anxa1a* was also significantly upregulated after 1,000mg/L NaCl pre-treatment but back to normal after 3,000mg/L NaCl pre-treatment.

### Effects of H_2_O_2_ and NaCl Pre-treatment During Egg Hatching on the Expression of Genes Involved in Antioxidant Capacity of Loach Larvae

**Figure 3:**
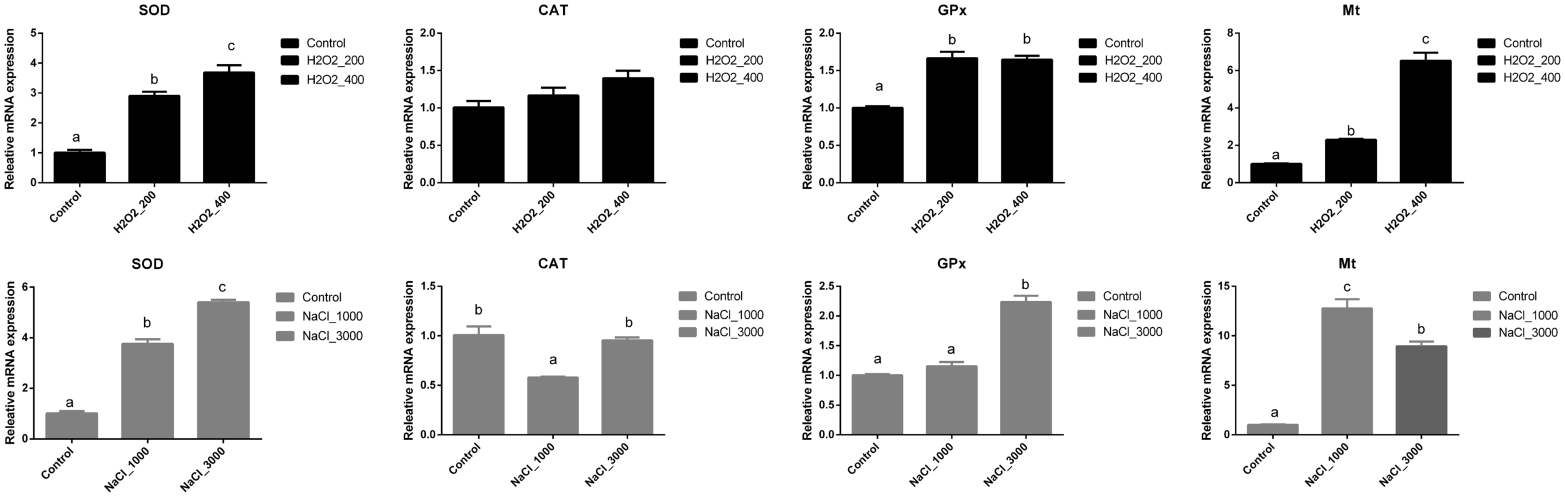
Effects of H_2_O_2_ (200, 400mg/ml) and NaCl (1,000, 3,000mg/ml) pre-treatment during egg hatching on the relative expression levels of genes involving antioxidant capacity, including *SOD, CAT, GPx,* and *Mt* in loach larvae.

The mRNA expression levels of genes involved in the antioxidant capacity of loach larvae after H_2_O_2_ and NaCl pre-treatment are shown in Figure 3. The expression levels of *SOD* and *Mt* were significantly upregulated with the increased dosage of H_2_O_2_, and the highest expression levels were both detected at 400mg/L H_2_O_2_ pre-treatment. The expression of *GPx* was also significantly upregulated after H_2_O_2_ pre-treatment; however, no significant differences were detected between two dosages. Additionally, the expression of *CAT* was not significantly affected by H_2_O_2_ pre-treatment.

The expression of *SOD* was significantly upregulated with the increased dosage of NaCl, and highest expression level was detected at 3,000mg/L NaCl pre-treatment. The expression of *GPx* was only significantly upregulated after 3,000mg/L NaCl pre-treatment. The expression of *Mt* was significantly upregulated by H_2_O_2_ pre-treatment, but the highest expression level was detected at 1,000mg/L NaCl pre-treatment. Additionally, 1,000mg/L NaCl pre-treatment significantly decreased the expression of *CAT*.

### Effects of H_2_O_2_ and NaCl Pre-treatment During Egg Hatching on the Expression of Genes Involved in Immune Response of Loach Larvae

**Figure 4:**
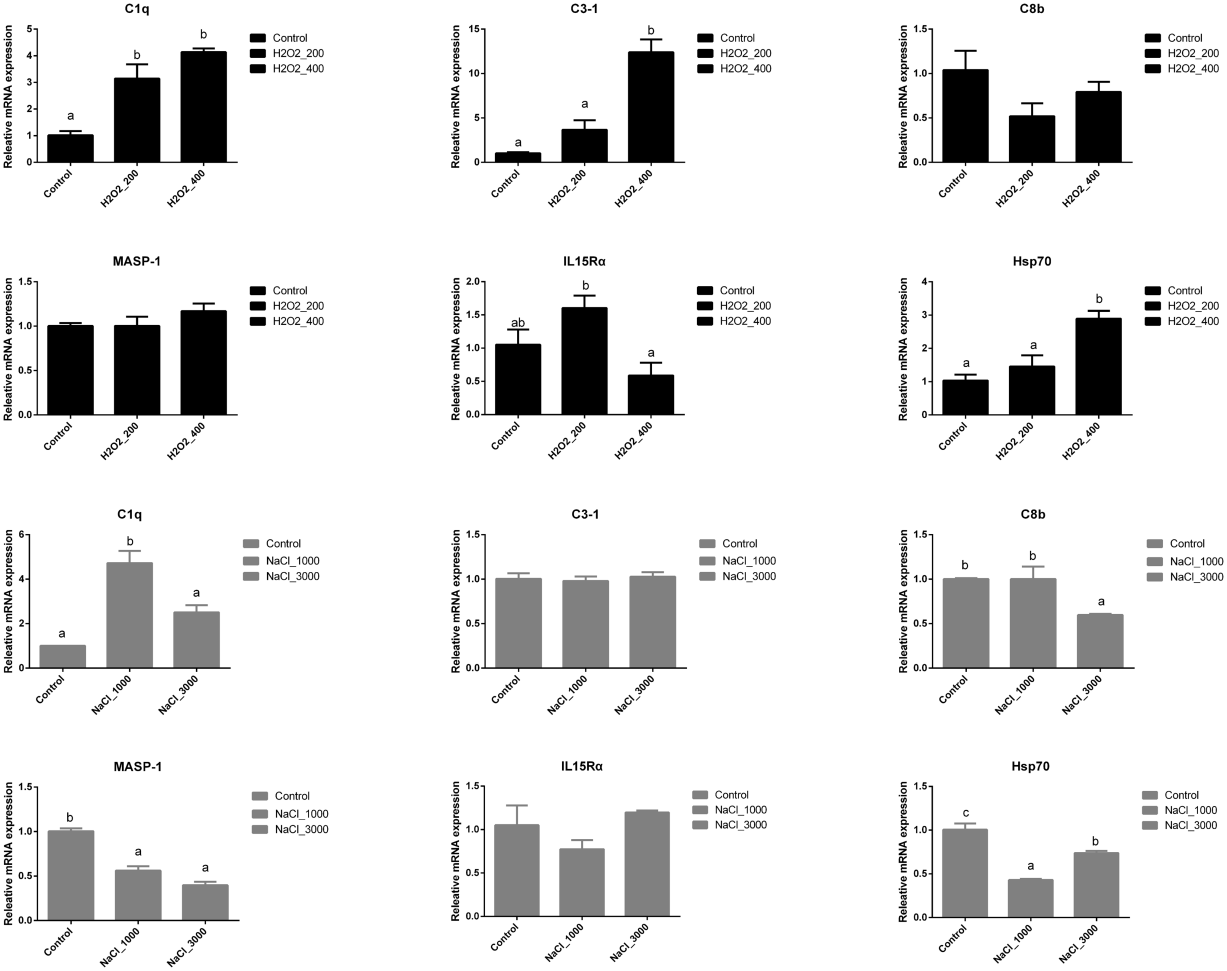
Effects of H_2_O_2_ (200, 400mg/ml) and NaCl (1,000, 3,000mg/ml) pre-treatment during egg hatching on the relative expression levels of genes involving immunity, including *C1q, C3-1, C8b, MASP-1, IL15Rα,* and *Hsp70* in loach larvae.

Figure 4 indicated the different expression levels of genes involved in the immune response of loach larvae after H_2_O_2_ and NaCl pre-treatment during egg hatching. The expression of *C1q* was also significantly upregulated after H_2_O_2_ pre-treatment; however, no significant differences were detected between two dosages. The expression of *C3-1* and *Hsp70* was significantly upregulated after 400mg/L H_2_O_2_ pre-treatment. The expression of *IL15Rα* was significantly higher in loach larvae after 200mg/L H_2_O_2_ pre-treatment than that after 400mg/L H_2_O_2_ pre-treatment. No significant influences of H_2_O_2_ pre-treatment were detected on the expression of *C8b* nor *MASP-1*.

The expression of *C1q* was also significantly upregulated after 1,000mg/L NaCl pre-treatment but back to normal after 3,000mg/L NaCl pre-treatment. The expression of *C8b* was significantly downregulated after 3,000mg/L NaCl pre-treatment. The expression of *MASP-1* was significantly downregulated after NaCl pre-treatment but no significant differences were detected between two dosages. The expression of *Hsp70* was significantly downregulated after NaCl pre-treatment, and the lowest expression level was detected after 1,000mg/L NaCl pre-treatment. No significant influences were detected on the expression of *C3-1* nor *IL15Rα* in loach larvae after NaCl pre-treatment.

### Effects of H_2_O_2_ and NaCl Pre-treatment During Egg Hatching on the Expression of Genes Involved in Lipid Metabolism of Loach Larvae

**Figure 5:**
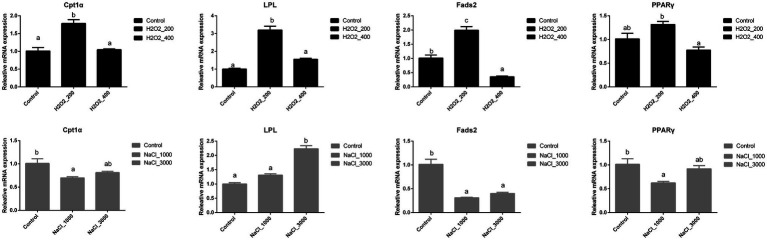
Effects of H_2_O_2_ (200, 400mg/ml) and NaCl (1,000, 3,000mg/ml) pre-treatment during egg hatching on the relative expression levels of genes involving lipid metabolism, including *Cpt1α, LPL, Fads2,* and *PPARγ* in loach larvae.

H_2_O_2_ and NaCl pre-treatment during egg hatching also significantly affected the expression of genes involved in the lipid metabolism of loach larvae (Figure 5). The expression of *Cpt1α*, *LPL*, and *Fads2* was significantly upregulated after 200mg/L H_2_O_2_ pre-treatment. The mRNA expression levels of *Cpt1α* and *LPL* went back to normal after 400mg/L H_2_O_2_ pre-treatment, while the expression of *Fads2* was even decreased after 400mg/L H_2_O_2_ pre-treatment. The expression of *PPARγ* was significantly higher in loach larvae after 200mg/L H_2_O_2_ pre-treatment than that after 400mg/L H_2_O_2_ pre-treatment.

The expression of *Cpt1α* and *PPARγ* was significantly downregulated after 1,000mg/L NaCl pre-treatment, but back to normal after 3,000mg/L NaCl pre-treatment. The genes expression level of *LPL* was significantly upregulated after 3,000mg/L NaCl pre-treatment. The expression of *Fads2* was significantly downregulated after NaCl pre-treatment, while no significant differences were detected between two NaCl dosages.

## Discussion

The disease prevention or control is of great importance to keep the healthy fish fry stocks (Subasinghe et al., 2000), so antibiotics and insecticides were traditionally applied during fish fry breeding. However, the applications of antibiotics and insecticides have been seriously restricted in many countries, including China, because they are not only highly toxic to humans and fish and not easy to be degraded in the environment, but also lead to the potential development of antibiotic resistance (Holmström et al., 2003; Cabello, 2006; Zhou et al., 2019; Shao et al., 2021). Fish vaccine is of great potential in the prevention of disease outbreaks; however, only eight fish vaccines have been licensed in China, which is far more from enough to main the continual development of aquaculture production in China (Wang et al., 2020). Especially in fish fry, there is no available fish vaccine nor mature vaccination route, which seriously restricts the stable fish fry stocks (Rojo-Cebreros et al., 2018). The safe and environmental-friendly drugs, including H_2_O_2_ and NaCl, have been tested in the fry hatching of many fish species (Magondu et al., 2011). H_2_O_2_ has been proved to promote the hatching rate of eggs in multiple fish species. For example, H_2_O_2_ treatment at 500–1,000ppm significantly increased hatching rates and controlled fungi in rainbow trout eggs (Schreier et al., 1996; Barnes et al., 1998). In channel catfish (*I. punctatus*), H_2_O_2_ treatment at low concentrations of 70–250mg/L significantly increased percent hatching of fish eggs (Small and Wolters, 2003), and later studies indicated that higher dosage of H_2_O_2_ (500 or 750mg/L) could also improve the percent hatching of channel catfish eggs (Rach et al., 2004). One study also compared the effects of H_2_O_2_ on eight species of warm- and cool-water fish eggs which identified the concentration of 1,000mg/L to be most effective in improving hatching rate (Rach et al., 1998). The unfertilized fish eggs are especially vulnerable to fungal infection from the family *Saprolegniaceae* (Post, 1987), which produces mycelia to facilitate spreading from the nonviable to the healthy eggs and cause egg mortality (Teresa Vega-Ramírez et al., 2013). In the present study, H_2_O_2_ treatment at 400mg/L significantly improved the hatchability and also decreased the egg mortality. However, the decreased egg mortality after 400mg/L treatment was not due to the inhibition of fungi as the fungi-induced egg mortality was even increased in 400mg/L H_2_O_2_ treatment. Four hundred milligram per liter H_2_O_2_ treatment might contribute to the other factors, including bacterial inhibition or water parameters protection. This was in accordance with studies in salmon (*Salmo salar*) as H_2_O_2_ concentration strongly affected salmon mortality, but did not alter mucous cell area or density, pre-adult lice removal efficiency, or the re-infection success of lice copepodids (Overton et al., 2018). Besides H_2_O_2_, NaCl has also been reported to affect the hatching rate of fish eggs; however, the effects varied depending on the dosage of NaCl and the fish species. Schnick (1988) reported that the 3,000ppm NaCl dip effectively removed protozoa from fish egg surfaces and limited any mycelial production that may lower egg hatching. Salt treatment at 0–5,000mg/L significantly improved egg hatching in channel catfish (Froelich and Engelhardt, 1996), and NaCl significantly improved the hatching rate of koi carp (*C. carpio haematopterus*) at 1,000 and 2,500mg/L for a 60min exposure duration but even toxic to the eggs at 5,000mg/L (Phelps and Walser, 1993). In the present study, NaCl treatment at 1,000 and 3,000mg/L did not significantly affect fish egg hatching rate nor the fry mortality. Moreover, the total mortality and fungi-induced mortality of loach eggs were also not significantly affected after NaCl treatment. However, 1,000mg/L NaCl treatment significantly inhibited the other factor-induced egg mortality excepting fungi. NaCl treatment showing no effects on the hatching performance of loach in the present study may be due to the test dosages of NaCl and the fish species. A much wider dosage range of NaCl during loach hatching could be tested in the future study.

Although plenty of studies have evaluated the influences of NaCl and H_2_O_2_ pre-treatment on the egg hatching of many fish species, little information is known about the influences of these pre-treatments on fish larvae health and nutrient metabolism. Recent studies have indicated that nutritional stimuli (quantity or quality of nutrients) and non-nutritional environmental stress experienced at critical periods of an organism’s life can result in permanent changes in postnatal growth potential, health, and metabolic status in animals including fish and shrimp (Burdge and Lillycrop, 2010; Hu et al., 2018). Moreover, temperature has also been reported to affect the liver transcriptome response of spotted seabass (*Lateolabrax maculatus*) induced by dietary protein level (Cai et al., 2020). Thus, the effects of NaCl and H_2_O_2_ during egg hatching on antioxidant capacity, immune responses, and lipid metabolism of loach larvae were systematically evaluated. In previous studies, the metabolic programming in aquatic has been mainly focused on carbohydrate metabolism due to the desired protein-sparing effects (Hu et al., 2018); however, lipid metabolism is also important and also serves the protein-sparing effect (Peng et al., 2019). Especially, fish larvae require much higher energy consumption for the rapid growth (Abi-Ayad and Kestemont, 1994; Gaon et al., 2021) and lipid serves as the most efficient nutrient for energy supply (Kupriyanova et al., 2021). In the present study, both the genes involved in lipid catabolism, such as *Cpt1α* and *LPL,* and genes involved in lipid anabolism, such as *Fads2,* along with the regulatory factor, *PPARγ*, were significantly upregulated in 200mg/L H_2_O_2_ pre-treatment group, but back to normal level at 400mg/L. However, most genes were downregulated by NaCl pre-treatment excepting *LPL* which was significantly upregulated after 3,000mg/L NaCl pre-treatment. This phenomenon has been reported in earlier studies which suggested that nutritional programming by dietary carbohydrates in European seabass larvae may not always be as expected (Zambonino-Infante et al., 2019). These differential results may result from different fish species, different stimulus patterns, and/or dosages.

In animals, the *ROS* play important roles in tissue homeostasis, cellular signaling, differentiation (Harris and DeNicola, 2020), and their levels are tightly regulated by cellular antioxidant system to prevent unwanted consequences (Pérez-Jiménez et al., 2017). However, oxidative stress will be generated when the balance between the production and neutralization of *ROS* is broken to favor the former, thus causing oxidative damage to proteins, nucleic acids and lipids, destroying important cellular processes and increasing mutations (Loro et al., 2012). Like in mammals, the cellular antioxidant defenses system in fish has been identified and proven to be functional during multiple situations which include *ROS* scavenging, oxidative stress protection, and attenuation of membrane lipid peroxidation (Hermans et al., 2007). Consequently, the major front-line antioxidant enzymes, such as *SOD* (neutralizes superoxide radicals to H_2_O_2_), *CAT,* and *GPx* (neutralizes H_2_O_2_ to water), and small non-protein antioxidants (scavenges all active oxygen species directly) work in a cascade to protect cells from oxidative stress (Ighodaro and Akinloye, 2018). Oxidative responses of both invertebrates and vertebrates under salinity challenges have been emphatically discussed. In juvenile olive flounders, the activities of *SOD*, *GST*, and *GSH* in the liver and gill were significantly affected by salinity (Kim et al., 2021). The activities of serum antioxidants, including *SOD, GPx, CAT,* and glutathione reductase (*GR*) in the spleen of European seabass after cold stress, were affected by salinity (Islam et al., 2020). Early studies have indicated the influences of environmental parameters including seawater acidification and cadmium on the antioxidant defense of flounder *P. olivaceus* larvae (Cui et al., 2020). In the present study, NaCl pre-treatment significantly induced the higher expression levels of *SOD*, *GPx*, and *Mt*, which is similar to previous studies in other juvenile fish and fish larvae. However, the expression level of *CAT* was not significantly upregulated but even decreased after 1,000mg/L NaCl pre-treatment. This is similar to earlier reports that, unlike *SOD*, no significant changes were observed in the mRNA expression or activity of *CAT* in the livers of *D. labrax* and *Chanos Chanos* under different salinity (Chang et al., 2021). H_2_O_2_, as a strong oxidant, can increase the intracellular *ROS* level and induce oxidative stress. However, the effects of H_2_O_2_ on fish antioxidant defense, including the levels of antioxidant enzymes (e.g., *SOD* and *CAT*) and nonenzymatic antioxidants (e.g., *GSH*), varied depending on the duration and dosage of H_2_O_2_ treatment. It has been reported that short and moderate H_2_O_2_ treatment stimulated the levels of the antioxidant enzymes, while chronic and severe H_2_O_2_ treatment impaired antioxidant defense system (Jia et al., 2021). In common carp (*C. carpio*), the oxidative stress-related genes, including *nrf2, gstα, sod, cat,* and/or *gpx1,* were upregulated in liver, gills, muscle, intestines, and/or kidney, but downregulated in heart after H_2_O_2_ exposure (Jia et al., 2020). In the brain and liver tissue of largemouth bass, 2.5mg/L sodium carbonate peroxyhydrate containing H_2_O_2_ as the active ingredient resulted in an increase of *SOD*, *CAT*, *GPX*, *GR*, and *GST* activity (Sinha et al., 2020). In the present study, the expression of *SOD*, *GPx,* and *Mt* in loach larvae was significantly increased after H_2_O_2_ pre-treatment. However, like the unaffected *CAT* expression during NaCl treatment, no significant changes were found on the expression of *CAT* in loach larvae after H_2_O_2_ pre-treatment. Thus, H_2_O_2_ (200 and 400mg/L) and NaCl (1,000 and 3,000mg/L) pre-treatment during egg hatching significantly stimulated the antioxidant defense system in loach larvae.

Besides antioxidant defense system, the immune system also protects fish against environmental stress and teleost is the first bony vertebrate to develop both innate and adaptive immunity. Salinity and H_2_O_2_ have been shown to affect the fish immune responses, for example, the immune responses of European seabass acclimatized after extreme ambient cold stress were significantly affected by different salinities (Islam et al., 2020) and transcriptome analysis also identified 100 differentially expressed genes involved in the immune system of common carp (*C. carpio*) after H_2_O_2_ exposure (Jia et al., 2021). Especially, the complement system, which is composed of more than 35 soluble plasma proteins, plays an essential role in alerting and clearing of potential pathogens and also contributes to the development of an acquired immune response (Ferreira and Cortes, 2021). The complement system of teleost fish, like that of higher vertebrates, can be activated through all three pathways of complement (Nakao et al., 2011). Complement 3 (*C3*), the key component in teleost, is present in several isoforms that are the products of different genes (Sunyer et al., 1996). The lectin pathway is initiated through the interaction of *MBL* (like *C1q*) and ficolins with sugar moieties expressed on the surface of many microorganisms. *C1q* has been cloned in multiple fish species, such as channel catfish *I. punctatus* (Li et al., 2012) and killifish *F. heteroclitus* (Kocabas et al., 2002). Moreover, *MASP1* has such a broad specificity and has significant substrates other than complement proteins (Hajela et al., 2002). Besides, *C8* is responsible for the formation of membrane attack complex (Liyanage et al., 2018). In the present study, H_2_O_2_ pre-treatment during egg hatching significantly induced the higher mRNA expression level of *C3-1* and *C1q* in loach larvae, but did not affect the mRNA expression level of *C8b* nor *MASP-1*. This was similar to previous study that the expression levels of complement *C3, C4*, and *C7* in the Atlantic salmon skin were significantly upregulated by 24-h exposure to H_2_O_2_ (Karlsen et al., 2021). NaCl pre-treatment only increased the mRNA expression level of *C1q*, but decreased the mRNA expression levels of *C8b* and *MASP-1*. The mRNA expression level of *C3-1* was not significantly affected during NaCl pre-treatment. Under the stimulation of inflammatory mediators, activation signals, and pathogenic infection, interleukin 15 (*IL15*) could transfer from the endoplasmic reticulum to cell membrane after binding with its receptor (*IL15R*) and control multiple process, including cell proliferation and inhibition of apoptosis (Chen et al., 2018). Additionally, *Hsps* has been shown to be an integral part of the cellular stress response pathways in fishes (Metzger et al., 2016) and widely used as biomarkers of exposure to environmental stressors (Mitra et al., 2018). In the present study, H_2_O_2_ pre-treatment induced the mRNA expression of *IL15Rα* at 200mg/L and *Hsp70* at 400mg/L, while NaCl pre-treatment decreased *Hsp70* expression at two dosages but did not affect the expression of *IL15Rα*.

Fish fry is rather fragile at the early development period and can be easily affected by the surrounding environment. As reported earlier, the newly hatched loach larva had a long straight intestinal tube with a very simple structure (Luo et al., 2016), and the effectiveness of drug pre-treatment on the fry could be monitored by evaluating the relative mRNA expression levels of early development-related genes. *Spon1b* was originally isolated from the developing embryonic floor plate of vertebrates and performs a positive function in nervous system development. A study in Japanese flounder showed that *spon1b* was maternally expressed with transcripts present from one-cell stage to hatching stage, peaking at tailbud stage (Hu et al., 2016). *IFT* sculpts the proteome of cilia and flagella and plays critical roles in cilia biogenesis, quality control, and signal transduction by delivering proteins to the growing ciliary tip and selectively transporting signaling molecules (Webb et al., 2020). *VEGFA* is required for the differentiation of endothelial cells (vasculogenesis) and for the sprouting of new capillaries (angiogenesis), and duplicated *VEGFA* in the zebrafish has been reported to mediate vascular development (Bahary et al., 2007). *Gdh* in the Antarctic fish *Chaenocephalus aceratus* has been reported to have relationship with cold adaptation (Ciardiello et al., 2000). *Anxa1a* also play a significant role in epimorphic regeneration of zebrafish caudal fin tissue (Quoseena et al., 2020). In zebrafish, *VIP*-like immunoreactive cells exist in the olfactory pit, the retina, and several regions of the brain at 24h post-fertilization (hpf) embryos (Mathieu et al., 2001). *PP1* and *PP2A* are proteins with major *Ser/Thr* protein phosphatase activity in eukaryotic cells and always interact with multiple proteins of diverse structure (regulatory subunits) with little substrate specificity; thus, they are a key regulator of cell development and oncogenic transformation (Dzulko et al., 2020). In the present study, excepting *anxa1a*, the expression levels of early development-related genes, including *spon1b, IFT22, VEGFAa, gdh, VIP, PP1,* and *PP2AB,* were significantly increased after 400mg/L H_2_O_2_ pre-treatment, which agrees well with the higher hatching rate in this group. However, although NaCl treatment increased their expression in loach larvae especially at 1,000mg/L, the hatching rate of loach was not significantly affected.

In all, our study indicated the long-time effects of H_2_O_2_ and NaCl pre-treatment during fish egg hatching on the health and metabolism of fish larvae. Besides the role in promoting egg hatchability, H_2_O_2_ pre-treatment at appropriate dosage also stimulated the antioxidant system and immune system of fish larvae, which could be linked to the good performance in fish early development.

## Data Availability Statement

The original contributions presented in the study are included in the article/supplementary material, further inquiries can be directed to the corresponding author.

## Ethics Statement

The animal study was reviewed and approved by Animal Experiment Committee of Huazhong Agricultural University.

## Author Contributions

QW designed and wrote the main context. MW conducted most experimental protocol. WX wrote the manuscript. JZ, SL, and ZS conducted the experimental analysis. FZ, WJ, and ZX supplied the relevant materials. All authors contributed to the article and approved the submitted version.

## Funding

This article was funded by National Natural Science Foundation of China (Grant Nos. 31802317 and 32172996).

## Conflict of Interest

The authors declare that the research was conducted in the absence of any commercial or financial relationships that could be construed as a potential conflict of interest.

## Publisher’s Note

All claims expressed in this article are solely those of the authors and do not necessarily represent those of their affiliated organizations, or those of the publisher, the editors and the reviewers. Any product that may be evaluated in this article, or claim that may be made by its manufacturer, is not guaranteed or endorsed by the publisher.
